# Therapeutic challenges in the management of osmotic demyelination syndrome: A case report of a favorable outcome from a tertiary center

**DOI:** 10.1097/MD.0000000000020283

**Published:** 2020-06-12

**Authors:** Mohamed Elshafei, Mohammed I. Danjuma, Rania El Tahir

**Affiliations:** a Hamad Medical Corporation (Hamad General Hospital); b Weill Cornell Medicine, Doha Qatar.

**Keywords:** demyelination, hyponatremia, osmotic

## Abstract

**Rationale:**

There is an increasing and compelling need for early recognition of features of osmotic demyelination syndrome (ODS), and a further attempt at correcting this even where presentation is late.

**Patient concerns:**

A 49-year-old male admitted into the emergency department with a complaint of lethargy and severe hyponatremia, with subsequent ODS supervening on initial attempts at correction.

**Diagnosis:**

Rapid rise in serum sodium concentration (121 mmol/L in 8 hours from a nadir of 101 mmol/L), concomitant deterioration in patient's conscious level support the diagnosis of ODS.

**Intervention:**

Concomitant administration of 5% dextrose water with desmopressin with a therapeutic objective of gradual relowering of serum sodium concentration.

**Outcomes:**

Significant improvement in patients’ conscious level and motor function with the commencement of sodium relowering therapy. The patient was eventually discharged home.

**Lessons:**

Regardless of the temporal profile of neurologic sequelae following ODS due to hyponatremia, its worthwhile attempting initial sodium relowering with dextrose 5% and desmopressin and then monitoring of biochemical and neurologic markers.

## 1 Introduction

The management of hyponatremia sometimes poses significant clinical challenges especially when this is complicated by osmotic fluid shifts.^[1]^ Of major concern is osmotic demyelination, which occurs as a result of the rapid correction of sodium (solute) deficit in patients with hyponatremia.^[1]^ Early recognition and treatment of this syndrome have a significant impact on near- and long-term morbidity and mortality.^[2,3]^ We present a case of severe iatrogenic hyponatremia with ODS with the favorable outcome despite late presentation with a novel concept of gradual sodium relowering using a combination of 5% dextrose water and desmopressin.

A 49-year-old male with a history of hypertension since few years, presented with weakness and fatigability associated with nausea and vomiting three to 4 times daily for 1 weak. In the last 2 days before admission, he started to feel dizzy and had gait imbalance.

There was no history of fever, abnormal sensation, focal weakness, or abnormal movements. However, he gave history suggestive of polydipsia but no polyuria and no history of use of diuretic medicine.

On presentation, he was conscious and alert (Glasgow coma scale [GCS] 15/15), blood pressure 128/80, hear rate 66/min. Cranial nerve examination was intact, and motor and sensory systems were normal. Apart from ataxic gait, there were no other features of cerebellar syndrome. Cardiorespiratory, neurologic, and abdominal system examinations were normal.

The initial metabolic panel showed severe hyponatremia (serum sodium Na was 102 mmol/L), serum osmolality, urine osmolality, and urine sodium were 228, 214, and 47 mmol/L, respectively. Table 1 gives the clinical time course of the patient stay in hospital vis-à-vis the kinetics of biochemical assays (including serum sodium). Other biochemical assays including the thyroid function test and assays for adrenal incompetence were normal. An emergent computed tomography brain scan done in Emergency department (ED) showed no acute brain insult. He also had a normal chest X-ray.

**Table 1:**
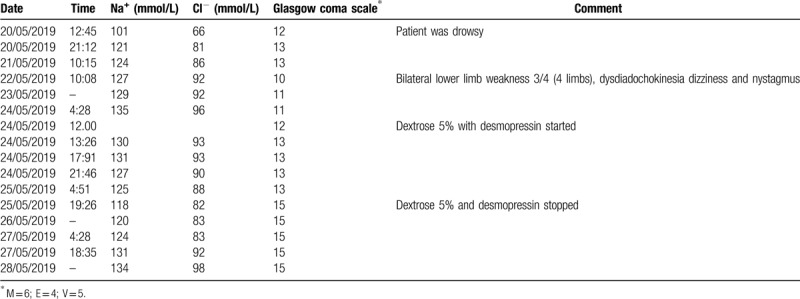
Temporal profile of biochemical markers as a function of the patient neurologic state at presentation and follow-up.

An initial impression of severe hypovolemic hyponatremia (most probably syndrome of inappropriate antidiuretic hormone [SIADH] secretion of yet to be a determined cause) with no neurologic deficit was made. The solute (Na^+^) deficit was estimated, and the patient commenced on fluid/solute resuscitation with hypertonic saline 2%. This resulted in inappropriately significant improvement in his serum sodium concentration (121 mmol/L in 8 hours from a nadir of 101 mmol/L). On attainment of serum sodium of 121 mmol/L, hypertonic saline was switched to normal saline (0.9%) until serum sodium reached the lower limit of normal (135 mmol/L) over 4 days as shown in Table 1.

On day 3, the patient started to feel nauseous and dizzy, could not walk or stand, and was vitally was stable but clinically was weak. He was globally week with a Power grade 3 in all 4 limbs (by medical research council grading of power). He also had features of the cerebellar syndrome including dysdiadochokinesia and nystagmus.

Consequent to the above development, a clinical impression of probable central pontine myelinolysis was made. This was predicated on the rapid correction of this gentleman's sodium deficit (especially its rapid rise from a nadir of 101 to 121 mmol/L within 8 hours). An emergent MRI brain was arranged, which showed no features of osmotic demyelination syndrome (ODS). Despite the negative radiologic features of ODS, the patient current presentation, and the already discussed circumstances surrounding his evidently over-enthusiastic sodium correction, strongly suggests the probability of early onset ODS. He was, therefore, commenced on dextrose 5% water infusion at a rate of 150 mL/h, and desmopressin 2 μg intravenously 6 hourly. This therapeutic strategy was aimed at gradual relowering of the serum sodium concentration with the view to prevent progression to established osmotic demyelination.

**Figure 1:**
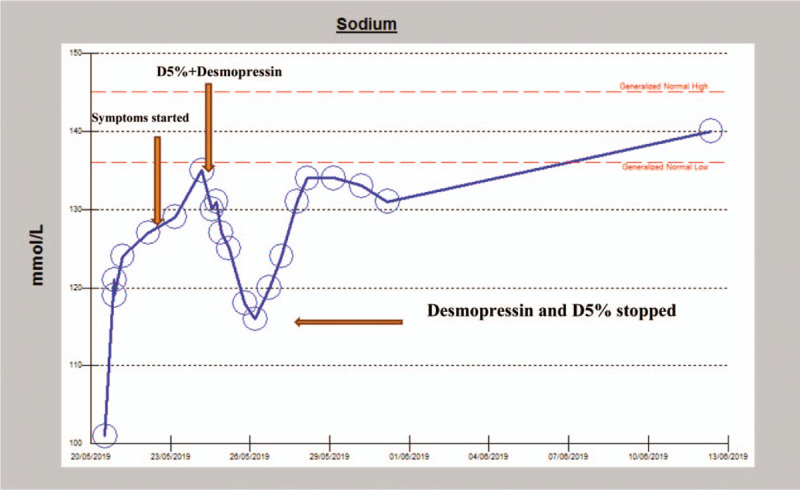
The temporal relationship between solute correction, and onset/resolution of neurologic symptoms.

Consequent to this, serum Na fell to 116 mmol/L over 34 hours. The patient motor function gradually improved after 2 days and was able to walk with no symptoms of dizziness. desmopressin and dextrose were stopped when Na reached 116 and was left to build up gradually and slowly until normal levels were attained over 4 days (as shown in Table 1, Fig. 1). On discharge, the patient was back to normal with no neurologic symptoms.

## 2 Discussion

To our knowledge this the 1st report of a successful attempt at the management of osmotic demyelination due to hyponatremia 48 hours after presentation. Osmotic demyelination contributes significantly to morbidity, and in some cases, mortality especially at the extremes of age (such as the very young and elderly).^[1,3]^ In patients with hyponatremia, ODS often supervenes as a result of rapid over-enthusiastic correction of solute deficit (10–12 mmol/L within 24 hours). Therefore, national and international clinical guidelines have suggested, and clinicians have adhered to this practice of gradual solute (Na^+^) replacement.^[2]^ Where ODS gets established, clinical manifestations are typically delayed for 2 to 6 days after an overly rapid elevation of the serum sodium concentration.^[3]^ The presentation of our patient is typical of this. Although he was drowsy at presentation due to hyponatremia, subsequent rapid sodium replacement (from 101 to 121 mmol/L) resulted in worsening of his neurologic features including progressive loss of consciousness (GCS of 10 = M = 4; E = 3, V = 3), global weakness with spasticity, and rigidity. The clinical impression of evolved ODS in our patient was very instructive, because though the rapid solute correction was very evident, the nonspecificity of his symptoms perhaps delayed the diagnosis. This then may have accounted for the seemingly delayed attempt at initiating solute correction (48 hours after the onset of symptoms). The steady but progressive response of our patient to treatment (dextrose 5% with desmopressin) was novel in our opinion. This so because though Croxson et al^[4]^ reported on a successful outcome for ODS management 36 hours after presentation, the favorable outcome of our case would appear to be the 1st report of its kind (48 hours after the onset of symptoms). It is also noteworthy that of all the previous reports, the median duration of presentation of ODS with favorable treatment outcomes was <10 hours.^[5–9]^ While it remains difficult to exactly identify the specific factor(s) that may have accounted for the favorable outcomes in our patient, we suspect that his young age, and lack of significant comorbidities may have had a role in this. It is also noteworthy that magnetic resonance imaging of our patient failed to demonstrate typical features of ODS in our patient despite overwhelming clinical parameters supporting this. It is pertinent to note that radiologic features of ODS usually take some time to evolve, in some reports extending up to 4 to 6 weeks.^[10,11]^

The brain's adaptation to change in the serum osmolality leads to development of neurologic symptoms if hyponatremia is corrected too rapidly. The combination of the adaptive decrease in intracellular osmolality and rapid increase in serum osmolality leads to rapid and excessive water movement out of the brain cells and intracellular fluid (ICF) volume depletion. Thus, too rapid correction of the serum sodium concentration can lead to an acute decrease in brain cell volume, which contributes to the pathogenesis of ODS, or central pontine myelinolysis. We hypothesize that relowering serum Na by desmopressin and free water infusion result in decreasing extracellular Osmolarity which is rapidly increased due to rapid correction and provide iso-osmolarity between brain cells and extracellular fluid which will prevent further water driving out of the brain cells.

Data in humans are limited to case reports that suggest benefit from early relowering of the serum sodium in patients who have developed symptoms of ODS.^[4,6–9,12]^ In these reports, relowering was initiated within a few hours after the onset of neurologic symptoms. Although there is no evidence of benefit in humans when relowering is begun more than 24 hours after the onset of ODS symptoms, we report this case for a patient who was commenced on relowering therapy after 48 hours of the symptoms with a favorable outcome. Secondly, it provides additional evidence of the effect of the relowering of serum sodium in the prevention of progression of demyelinosis.

Our report is limited by the usual setbacks associated with attempting treatment without clinical guidelines support.

We could conclude that our report is the first case of a favorable outcome following the management of late presentation of ODS through gradual sodium relowering (more than 48 hours of the onset of symptoms). This is likely to add to the increasing reports of attempts at management of osmotic demyelination despite lack of robust adequate explicit clinical guidelines support.

Given our case, two important lessons need to be addressed by clinicians, Firstly, there is a need for strict adherence to the protocol of gradual solute (Na^+^) replacement in patients with symptomatic hyponatremia. Secondly, Regardless of the temporal profile of neurologic sequelae following ODS due to hyponatremia, it's worthwhile attempting initial sodium relowering with dextrose 5% and desmopressin and then monitoring of biochemical and neurologic markers.

## Author contributions

**Conceptualization:** Mohamed elshafei, Rania El Tahir.

**Data curation:** Mohamed elshafei.

**Writing – original draft:** Mohamed elshafei, Mohammed Danjuma, Rania El Tahir.

**Writing – review & editing:** Mohamed elshafei, Mohammed Danjuma, Rania El Tahir.
